# Seroreactivity against Marburg or related filoviruses in West and Central Africa

**DOI:** 10.1080/22221751.2019.1709563

**Published:** 2020-01-08

**Authors:** Imke Steffen, Kai Lu, Nicole A. Hoff, Prime Mulembakani, Emile Okitolonda Wemakoy, Jean-Jacques Muyembe-Tamfum, Nicaise Ndembi, Catherine A. Brennan, John Hackett, William M. Switzer, Sentob Saragosti, Guy O. Mbensa, Syria Laperche, Anne W. Rimoin, Graham Simmons

**Affiliations:** a Vitalant Research Institute, San Francisco, CA, USA; b Department of Laboratory Medicine, University of California San Francisco, San Francisco, CA, USA; c Department of Epidemiology, School of Public Health, University of California Los Angeles, Los Angeles, CA, USA; d Kinshasa School of Public Health, University of Kinshasa, Kinshasa, Democratic Republic of the Congo; e Institut National de Recherche Biomedicale, Kinshasa, Democratic Republic of the Congo; f Institute of Human Virology, Abuja, Nigeria; g Abbott Diagnostics, Abbott Park, IL, USA; h Centers for Disease Control and Prevention, Atlanta, GA, USA; i INSERM U941, Paris, France; j Centre National de Transfusion Sanguine, Kinshasa, Democratic Republic of Congo; k Institut National de la Transfusion Sanguine, Paris, France

**Keywords:** Filoviruses, Marburgvirus, hemorrhagic fever virus, serology, prevalence, Cameroon, Ghana

## Abstract

A serological survey of 2,430 archived serum samples collected between 1997 and 2012 was conducted to retrospectively determine the prevalence of Marburg virus in five African countries. Serum samples were screened for neutralizing antibodies in a pseudotype micro-neutralization assay and confirmed by enzyme-linked immunosorbent assay (ELISA). Surprisingly, a seroprevalence for Marburg virus of 7.5 and 6.3% was found in Cameroon and Ghana, respectively, suggesting the circulation of filoviruses or related viruses outside of known endemic areas that remain undetected by current surveillance efforts. However, due to the lack of validated assays and appropriate positive controls, these results must be considered preliminary.

Filoviruses, including Ebola and Marburg viruses, cause outbreaks of viral disease with a broad range of clinical symptoms, including hemorrhagic fever, and high fatality rates in humans and non-human primates in Central Africa [1]. Increasing evidence points to an association of filoviruses with fruit bats as at least one natural reservoir host. Marburg virus has been isolated from *Rousettus aegyptiacus* bats, and viral RNA and antibodies have been detected in several other species [2,3]. Moreover, Marburg virus spillover infections to humans are often linked to entering caves or mines housing large bat populations [2,4]. Prior to 2014, it was assumed that filoviruses were restricted to small pockets in Central Africa where they sporadically made the jump from animal reservoirs to local human populations. However, the emergence of Ebola virus in West Africa in 2014 and its subsequent spread to densely populated areas was surprising and led to missed opportunities to control spread in the early phase of the outbreak [5]. This outbreak directed us and others to more closely investigate the geographical distribution and epidemiology of filoviruses in animals and humans across wider parts of Central Africa. One of our recent studies aimed to determine the serologic prevalence of Ebola virus in human populations of five West and Central African countries [6] (Table 1). We tested 2,430 serum samples collected initially for other epidemiologic studies for antibodies against three major Ebola virus antigens (glycoprotein (GP), nucleoprotein (NP) and matrix protein (VP40)) using three different assay formats, including neutralization, ELISA and a luciferase immunoprecipitation system (LIPS) [6]. Our findings suggested a low overall prevalence of 2–3.5% in known endemic countries, such as the Republic of Congo (ROC) and the Democratic Republic of Congo (DRC). Additionally, a small percentage of samples (1.3%, *n* = 160) from Southern Cameroon were reactive with two or more Ebola virus antigens indicating a potential risk for filovirus exposure in this non-endemic region of Africa [6]. To scrutinize these results and to test for cross-reactivity with antigens of other related viruses, we additionally screened all samples for antibodies against the Marburg virus glycoprotein (MARV-GP) in a high-throughput pseudotype neutralization assay (Figure 1C–J). Pseudotypes carrying the glycoprotein (GP) of the unrelated South American arenavirus Machupo virus (MACV) were included as a specificity control. Samples that reduced the infectivity of MARV-GP pseudotypes by more than 50% with no change in infectivity of control pseudotypes were titrated over a broader range of concentrations to confirm neutralizing activity. Reactive samples were additionally tested in a commercially available ELISA for the detection of anti-MARV-GP antibodies (Figure 1B).

**Figure 1.**
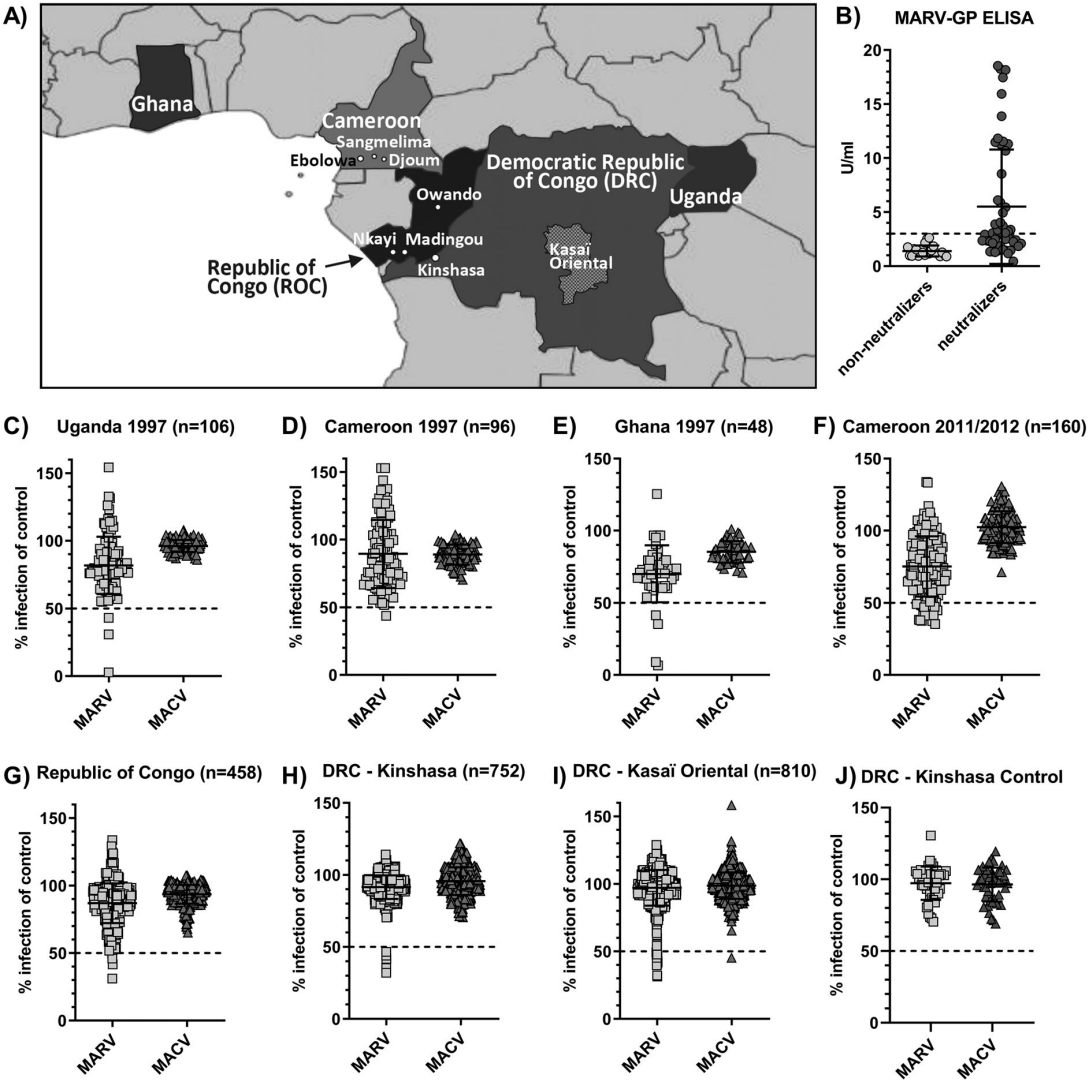
Serological screening of 2,430 serum samples from five African countries for antibodies against the Marburgvirus glycoprotein (MARV-GP). Close-up map of Central Africa with approximate location of relevant towns, cities and districts (A). Results of serum neutralization at 1:50 dilution of MARV-GP or Machupo virus glycoprotein (MACV-GP) pseudotyped HIV with samples from Uganda (C), Cameroon (D, F), Ghana (E), Republic of Congo (G), Kinshasa, DRC (H), Kasaï Oriental, DRC (I) and negative control samples from Kinshasa, DRC (J). Confirmatory ELISA results for detection of anti-MARV-GP antibodies in 51 neutralizing samples compared to 22 randomly selected non-neutralizing samples across all sample sets (B).

**Table 1.** Summary of sample origin, numbers, risk group and testing results in the Marburgvirus (MARV) neutralization assay (MARV Neut) and MARV-glycoprotein (GP) ELISA.

Country	Location	Risk group	Collection period	Sample Number	MARV Neut	MARV ELISA	MARV double reactive	single + double reactive
Uganda	unknown	AIDS	1997	106	3	3/3	3	3
		(100% HIV-positive)			2.8%	100%	2.8%	2.8%
Cameroon	unknown	AIDS	1997	96	1	0	0	1
		(100% HIV-positive)			1.0%	0.0%	0.0%	1.0%
Ghana	unknown	AIDS	1997	48	4	3/4	3	4
		(100% HIV-positive)			8.3%	75.0%	6.3%	8.3%
Cameroon	all locations	illness of unknown etiology	2011–2012	160	18	12/18	12	18
					11.3%	66.7%	7.5%	11.3%
Cameroon	Djoum	illness of unknown etiology	2011–2012	35	0	0	0	0
					0.0%	0.0%	0.0%	0.0%
Cameroon	Ebolowa	illness of unknown etiology	2011–2012	80	13	8/13	8	13
					16.3%	61.5%	10.0%	16.3%
Cameroon	Sangmelima	illness of unknown etiology	2011–2012	45	5	4/5	4	5
					11.1%	80.0%	8.9%	11.1%
ROC	all locations	HIV surveillance	1999	458	4	4/4	4	4
		(3.5% HIV-positive)			0.9%	100%	0.9%	0.9%
ROC	Madingou	HIV surveillance	1999	149	1	1/1	1	1
		(4.7% HIV-positive)			0.7%	100%	0.7%	0.7%
ROC	Nkayi	HIV surveillance	1999	149	1	1/1	1	1
		(2.7% HIV-positive)			0.7%	100%	0.7%	0.7%
ROC	Owando	HIV surveillance	1999	160	2	2/2	2	2
		(3.1% HIV-positive)			1.3%	100%	1.3%	1.3%
DRC	Kinshasa	blood donors	2011–2012	752	5	2/5	2	5
					0.7%	40.0%	0.3%	0.7%
DRC	Kasaï Oriental	monkeypox surveillance	2007	810	16	2/16	2	16
					2.0%	12.5%	0.2%	2.0%
Total			1997–2012	2430	51	26/51	26	51
					2.1%	51.0%	1.1%	2.1%

Samples were considered MARV reactive if they gave positive results in both assays (highlighted column), whereas the last column includes the cumulative number of samples that were reactive in one or both assays to represent the upper limit of the estimated seroprevalence.

The viral glycoproteins were expressed from a pCAGGS expression plasmid. Pseudotype viruses were generated in HEK293T cells as previously described [7] with 30 μg MARV-GP or MACV-GP expression plasmids and 10 μg pNL-Luciferase or pNL-Renilla HIV reporter backbones (*NIH AIDS Reagent Program, Division of AIDS, NIAID, NIH;* catalog #3418) [8], respectively. Neutralization assays were performed with human muscle rhabdomyosarcoma (RD) cells. Heat-inactivated human sera were diluted in culture medium, mixed with viral inoculum (containing a mix of both MARV-GP and MACV-GP pseudotypes encoding the two different luciferase enzymes) and incubated at room temperature for 1 h. Final serum dilutions of 1:50 and 1:500 were used in the high-throughput screen, while dilutions ranging from 1:10 to 1:31,250 were used in titrations. Subsequently, 30,000 RD cells/well were added and plates were incubated at 37°C for 48 h. All infections were performed in duplicate and each plate contained identical controls, including uninfected cells, cells infected in the absence of serum, and cells infected with virus incubated with negative control serum from US blood donors. Cells were lysed and luciferase activities in cell lysates were measured with the Dual-Glo Luciferase Assay System (Promega). Infection rates in the presence of serum samples were expressed as a percentage of infection in the presence of negative control serum.

ELISA kits for the detection of human anti-Marburg virus glycoprotein (GP) IgG were purchased from *Alpha Diagnostics International* (catalog # AE-322620-1) and were performed following the manufacturer’s instructions at 1:200 sample dilutions with absorbance read at 450 nm. Four calibrators are included with the ELISA kit. Assay specifications require calibrator OD values to be plotted against concentration and a linear fit is applied to the data. An *R*^2^ greater than .90 must be observed for the plate to pass quality testing. As the cut-off value recommended by the manufacturer of 1.0 U/ml generated unrealistically high positivity rates, we used samples from a similar locality to determine background reactivity. The cut-off value for the MARV-GP ELISA was ultimately defined to be 2.94 U/ml based on the average plus three standard deviations of the background signal in 47 samples from Kinshasa, DRC, that were presumed negative on the basis of low reactivity in both assays (Figure 1J). Serum samples reactive in both MARV-GP specific assays were considered seroreactive. Prevalence rates were calculated based on the number of seroreactive specimens.

Testing of HIV-positive samples collected in 1997 in Cameroon, Uganda and Ghana identified 8 neutralizing specimens, one in Cameroon (1.0%, *n* = 96), three in Uganda (2.8%, *n* = 106) and four in Ghana (8.3%, *n* = 48) (Table 1, Figure 1C–E). While the neutralizing sample in Cameroon was not confirmed by MARV-GP ELISA, all three neutralizing samples from Uganda and three of four neutralizing samples from Ghana were also reactive in the MARV-GP ELISA (Table 1, Figure 1B). This resulted in MARV seroprevalence rates of 2.8% in Uganda and 6.3% in Ghana (Table 1). Surprisingly, 18 samples collected in Cameroon in 2011/2012 (11.3%, *n* = 160) showed neutralizing activity against MARV pseudotypes (Table 1, Figure 1F), of which 13 originated from Ebolowa and five from Sangmelima (Figure 1A, Table 1). Twelve neutralizing samples (eight from Ebolowa and four from Sangmelima) were confirmed in the MARV-GP ELISA (Table 1, Figure 1B), resulting in MARV seroprevalence rates of 7.5% in Cameroon overall and local rates of 10.0% in Ebolowa and 8.9% in Sangmelima based on the 2011/2012 samples included in our study (Figure 1A, Table 1). Four samples from the ROC (0.9%, *n* = 458) were found to neutralize MARV pseudotypes (Table 1, Figure 1G), including two from Owando and one each from Nkayi and Madingou (Table 1). All four samples were confirmed by MARV-GP ELISA (Table 1, Figure 1B), resulting in an overall MARV seroprevalence of 0.9% in the ROC (Table 1). Of the ROC samples, 3.5% were HIV-positive. However, no correlation between HIV status and MARV seroreactivity could be determined. Five blood donor samples from Kinshasa, DRC (0.7%, *n* = 752) contained MARV pseudotype-neutralizing antibodies (Table 1, Figure 1H). Among these, two were confirmed by MARV-GP ELISA (Table 1, Figure 1B) resulting in 0.3% serological prevalence of MARV in this population (Table 1). In contrast, 16 samples from Kasaï Oriental in the central DRC (2.0%, *n* = 810) showed neutralizing activity specific for MARV pseudotypes (Table 1, Figure 1I). However, only two of 16 neutralizing specimens could be confirmed by the MARV-GP ELISA testing (Table 1, Figure 1B) for a specific MARV seroprevalence of 0.2% in Kasaï Oriental (Table 1). Where available, demographic data was tested for possible correlation with MARV seroreactivity within the individual sample sets. However, no pattern for increased (or reduced) risk of possible MARV exposure emerged. The quantification of MARV-GP antibodies by ELISA did not correlate with neutralization activity. Only one sample from Kasaï Oriental showed cross-reactivity against Ebola and Marburg virus glycoproteins with weak neutralizing activity against both viruses. None of the Ebolavirus reactive samples tested positive in the MARV-GP ELISA, suggesting sufficient specificity to distinguish between two related filoviruses. However, the sensitivity of the assay has not been systematically determined and it remains unclear if any of the neutralizing, but ELISA-negative samples were due to high background signals. For the present study we chose to select rather stringent assay cut-offs to avoid an overestimation of the resulting seroprevalence.

The reactivity of a small number of serum samples in our study against MARV-GP in pseudotype neutralization and ELISA may suggest a wider geographical range of MARV or related filoviruses and their natural reservoir hosts across different regions of Africa. However, in contrast to our study on Ebolavirus seroprevalence, confirmed MARV-reactive samples were not available to us for inclusion in our MARV neutralization assays as positive controls. For this reason, our assays could not be validated and the results shown here should be considered preliminary. In light of recent studies on the presence of MARV in West Africa [9], we see value in sharing our data nevertheless. In fact, a recent survey of 675 human serum samples from Sierra Leone found a prevalence of anti-MARV antibodies of 10.7% [10]. MARV RNA has recently been detected in bats in Sierra Leone (unpublished data communicated by Drs. Brian Bird and Jonathan Towner [9]) and serological evidence for MARV infection of several bat species has been found in the Northern ROC and Gabon, direct southern neighbours of Cameroon [11]. Furthermore, a recent study modelling the risk of zoonotic MARV transmission across Sub-Saharan Africa found a substantial threat for MARV transmission in Cameroon and identified Southern Cameroon as a beneficial target site for future surveillance efforts [12]. Together these studies highlight an increasing body of evidence for the presence of filoviruses in previously unrecognized regions of Africa.

## References

[1] Murray MJ. Ebola virus disease: a review of its past and present. Anesth Analg. 2015;121:798–809. doi: 10.1213/ANE.0000000000000866 26287303

[2] Amman BR, Carroll SA, Reed ZD, et al. Seasonal pulses of Marburg virus circulation in juvenile Rousettus aegyptiacus bats coincide with periods of increased risk of human infection. PLoS Pathog. 2012;8:e1002877. doi: 10.1371/journal.ppat.1002877 23055920 PMC3464226

[3] Swanepoel R, Smit SB, Rollin PE, et al. Studies of reservoir hosts for Marburg virus. Emerging Infect. Dis.. 2007;13:1847–1851. doi: 10.3201/eid1312.071115 PMC2876776 18258034

[4] Bausch DG, Nichol ST, Muyembe-Tamfum JJ, et al. Marburg hemorrhagic fever associated with multiple genetic lineages of virus. N Engl J Med. 2006;355:909–919. doi: 10.1056/NEJMoa051465 16943403

[5] Piot P, Muyembe JJ, Edmunds WJ. Ebola in West Africa: from disease outbreak to humanitarian crisis. Lancet Infect Dis. 2014;14:1034–1035. doi: 10.1016/S1473-3099(14)70956-9 25282665

[6] Steffen I, et al. Serologic prevalence of Ebola virus in equatorial Africa. Emerging Infect. Dis.. 2019;25:911–918. doi: 10.3201/eid2505.180115 PMC6478206 31002071

[7] Salvador B, Sexton NR, Carrion R, et al. Filoviruses utilize glycosaminoglycans for their attachment to target cells. J Virol. 2013;87:3295–3304. doi: 10.1128/JVI.01621-12 23302881 PMC3592117

[8] Zhou Y, Steffen I, Montalvo L, et al. Development and application of a high-throughput microneutralization assay: lack of xenotropic murine leukemia virus-related virus and/or murine leukemia virus detection in blood donors. Transfusion. 2012;52:332–342. doi: 10.1111/j.1537-2995.2011.03519.x 22239212 PMC3299481

[9] Centers for Disease Control and Prevention. Deadly Marburg virus found in Sierra Leone bats. 12-21-2018 [cited 2019 Aug 14]. Available from: https://www.cdc.gov/media/releases/2018/p1220-marburg-found-in-bats.html

[10] O’Hearn AE, Voorhees MA, Fetterer DP, et al. Serosurveillance of viral pathogens circulating in West Africa. Virol J. 2016;13:163. doi: 10.1186/s12985-016-0621-4 27716429 PMC5048616

[11] Pourrut X, Souris M, Towner JS, et al. Large serological survey showing cocirculation of Ebola and Marburg viruses in Gabonese bat populations, and a high seroprevalence of both viruses in Rousettus aegyptiacus. BMC Infect Dis. 2009;9:159. doi: 10.1186/1471-2334-9-159 19785757 PMC2761397

[12] Pigott DM, Golding N, Mylne A, et al. Mapping the zoonotic niche of Marburg virus disease in Africa. Trans R Soc Trop Med Hyg. 2015;109:366–378. doi: 10.1093/trstmh/trv024 25820266 PMC4447827

